# Microwave resonator array with liquid metal selection for narrow band material sensing

**DOI:** 10.1038/s41598-021-88145-3

**Published:** 2021-04-21

**Authors:** Benjamin D. Wiltshire, Md Abdur Rafi, Mohammad H. Zarifi

**Affiliations:** Okanagan Microelectronics and Gigahertz Applications Laboratory, School of Engineering, University of British Columbia, Kelowna, BC V1V 1V7 Canada

**Keywords:** Electrical and electronic engineering, Electronic and spintronic devices

## Abstract

A microwave resonator array is integrated with liquid metal to select an individual resonator response within a resonator array, enabling simple and accurate analysis for dielectric sensing. Galinstan, a liquid metal, acts as a multiplexer by inducing a capacitive load to the nearby resonator, lowering its resonant frequency, and thereby isolating its resonant response from other resonators in the array. The liquid metal could be positioned within a fluidic channel to be above any of the resonators, which tuned the resonant frequency from 3.9 to 3.3 GHz where it can be analyzed individually. The resonators showed a consistent response to liquid metal tuning, with tuning error measured below 30 MHz (5%). The sensor also exhibited stable sensitivity to test materials placed on the selected resonator, with a maximum resonant frequency shift of 300 MHz for a dielectric test material (ε = 10.2) and almost no variation in resonant amplitude. The selected resonant response was only sensitive to materials on the selected resonator, and was unaffected by test materials, even when placed on other resonators. The presented design enabled robust and accurate detection of materials using planar microwave resonators that can be controlled at a user’s convenience, specifically for use in systems where multiple parameters or system settings may need to be individually determined.

## Introduction

Planar microwave resonators have high potential in the world of smart sensors due to their robust, accurate, and contactless sensing capabilities^1,2^. Operating by detecting the dielectric permittivity and loss tangent of a nearby material^3–5^, planar microwave resonator sensors have demonstrated potential for leak detection in pipelines^6,7^, water quality monitoring^1,8^, glucose detection and biosensing^9–11^, and hazardous gas detection^12–14^ in various applications. Due to success in previous studies for sensing a wide variety of stimuli, the design of a sensor or sensor array that can measure many environmental variables at once, and in a cost-effective manner, has attained scientific interest^15–17^.

In microwave sensing applications, a typical split ring resonator sensor is fabricated on a substrate and connected with readout circuitry such as a vector network analyzer (VNA) to measure the variation in its resonant amplitude and frequency as the permittivity of the local environment changes. Primarily, research has gone towards implementing single resonators designed to detect the signatures of molecules or compounds, but this is an expensive practice that underutilizes the readout circuitry capabilities. Therefore, it is important to investigate ways to effectively condense multiple sensors down to a single sensor, either through multiplexing, machine learning analysis, or utilizing active circuit components. This can simplify the design, minimize the ecological footprint, and reduce costs^18–20^. Recently, a study by Ansari et al.^15^ implemented multiple split ring resonators on a single substrate to identify different materials’ dielectric properties. The sensor was calibrated to measure the dielectric properties of multiple materials simultaneously, but a wide frequency bandwidth of 1–7 GHz was necessary to prevent overlap between the resonant responses. In consideration of this design and the wide frequency range required for multivariable sensing, methods of selecting sensors by tuning their resonant frequency in a controlled manner has been investigated by utilizing varactors, MEMS devices, and liquid metal^21–23^. Liquid metal in particular is an interesting design choice, and in a study by Sadasivan et al.^24^ it was used in a cavity resonator to tune the resonant frequency by more than a full octave. Liquid metal is beneficial because it can be easily controlled and can be implemented in flexible designs, it also requires no power consumption or complex fabrication, and it can offer continuous tuning of the resonant frequency by introducing new coupling interactions^21,25–27^. Liquid metals such as EGaIn and Galinstan have been widely studied to determine their electrical and rheological properties which make them excellent candidates for tuning a high frequency response^28–30^. In a previous study^25^, liquid metal was shown to change a modified split ring resonator’s resonant frequency by up to 800 MHz, indicating that it could be a valuable method to passively tune a resonator response in a simple and precise way.

In this work, an array of planar microwave resonators is integrated with a fluidic channel which utilizes liquid metal to configure the resonant frequency of a resonator for sensing operation. Each resonator within the array, when not coupled to the liquid metal, operated at the same resonant frequency in the S-band due to their identical dimensions, but individual resonators could have their signals selectively tuned with liquid metal which altered their resonant frequency. In this way, a tuned resonator in the array could have its response analyzed without signal overlap with the untuned resonators, enabling accurate sensing without requiring a large frequency range. Investigations were performed to characterize the resonator array’s response to different sensing configurations as well as the individual resonator sensitivity and accuracy.

## Methods

**Figure 1:**
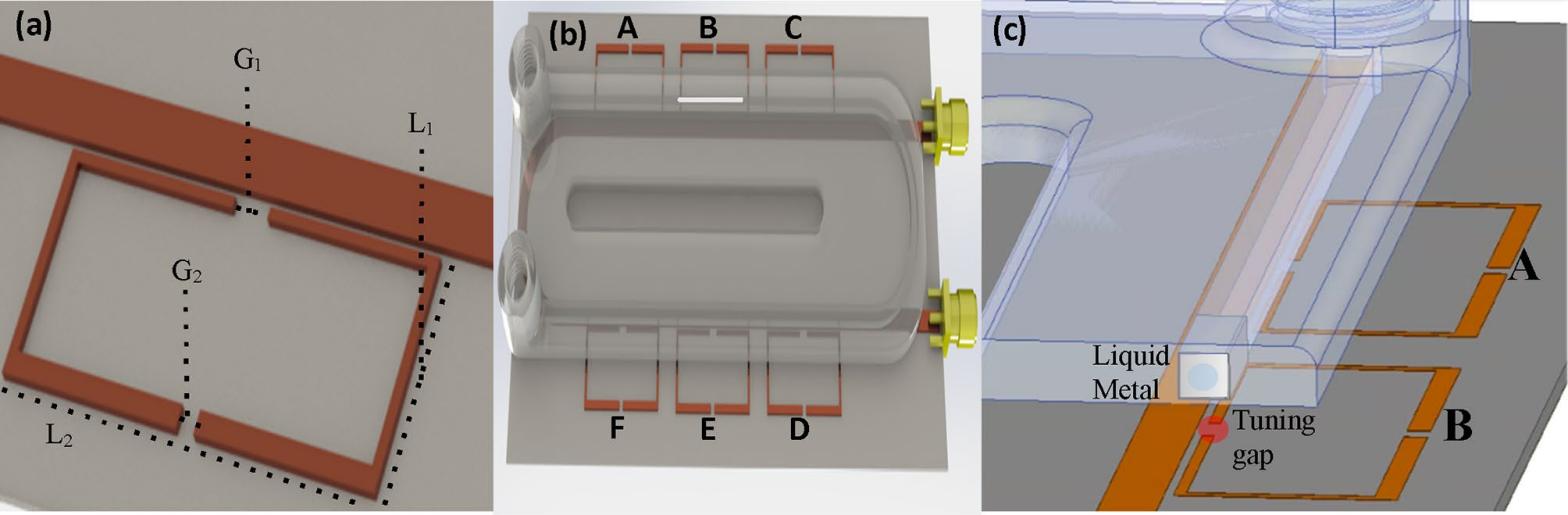
(**a**) The individual resonator dimensions, where G_1_ = 2 mm, G_2_ = 1 mm, L_1_ = 9.5 mm, and L_2_ = 21 mm, (**b**) the designed sensor array and microfluidic channel with liquid metal positioned at resonator B and (**c**) a cropped resonator showing the liquid metal and resonator coupling alignment. Images were rendered by *Solidworks* software, 2019 edition (https://www.solidworks.com).

In order to investigate the potential of liquid metal to provide selection control of particular resonators, an array of six modified split ring resonators with identical dimensions was designed and implemented using high frequency structure simulator (*HFSS*, version 19.0, https://www.ansys.com) software (Fig. 1a). Due to the matching size and shape of the resonators, similar coupling was observed between each individual ring resonator and the transmission line. A fluidic channel with a cross section of 0.5 × 1.0 mm was positioned above the array to ensure precise transportation of Galinstan, a liquid metal compound of gallium, indium, and tin. Hydrocal oil was used to prevent the liquid metal from sticking to the polymer walls and to improve its surface stability as it moved through the fluidic channel. Because of the channel’s large cross-sectional area (0.5 mm^2^) and possibly due to the naphthenic acid within the oil preventing gallium oxide accumulation on the surface of the Galinstan, the liquid metal did not adhere to the walls and flowed easily through the channel at a rate of 0.2 cm per second^28^. The liquid metal in the channel was separated 50 µm from the substrate and did not directly contact the resonator to prevent unwanted corrosion or electrical drift. Figure 1a presents the individual resonator structure and dimensions while Fig. 1b illustrates the overall resonator array with the attached fluidic channel to transport the liquid metal with Fig. 1c showing the position of coupling between the liquid metal and the resonator tuning gap.

As shown in Fig. 1a, each resonator had two gaps; one was used as a selection gap (G_1_), where liquid metal was used to tune the resonant frequency of the selected resonator, while the second gap was used as the sensitive region for material detection and sensing purposes (G_2_). The selecting ability of the liquid metal can be understood by modelling the tuning capacitance, *C*_s_, of an introduced planar metal above the circuit as in Eq. (1).

1 $$C_{s} = \frac{\varepsilon Wl}{d}$$

where *ε* is the permittivity of the fluidic channel, *W* is the width of the channel, *l* is the liquid metal length, and *d* is the channel wall thickness. *C*_*s*_ is the capacitance introduced by the liquid metal stub. The complete circuit model is described in a previous work^25^ and demonstrated that introducing *C*_*s*_ to an individual resonator can cause large changes in the resonant frequency that allow its S_21_ response to be analyzed without overlap between the other signals.

**Figure 2:**
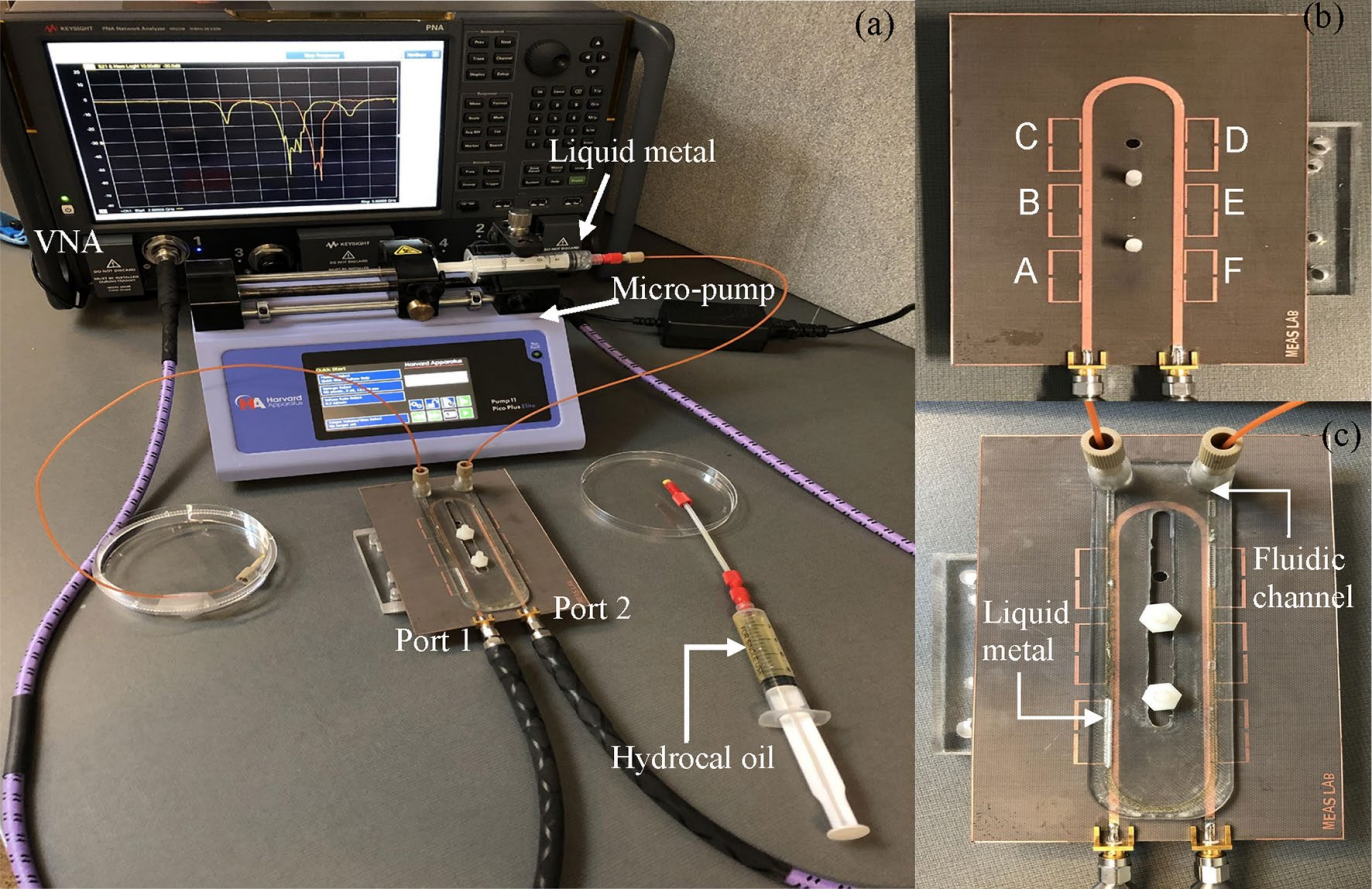
(**a**) The experimental setup with the required equipment labelled, (**b**) the resonator array with each resonator distinctly labelled for analysis (A through F), and (**c**) the resonator array with the integrated microfluidic channel and Galinstan aligned over resonator *A*.

The sensor array was fabricated on a Rogers RT/duroid 5880 substrate with thickness of 0.79 mm, the substrate permittivity and loss tangent were 2.20 and 0.0009. The fluidic channel was 3D printed with a copolyester material with a permittivity of 2.1 and loss tangent of 0.008. The channel and the substrate were mechanically attached to ensure consistent measurements. The experimental setup and the fabricated resonator and channel are shown in Fig. 2. Measurements were performed with a N5222B Vector Network Analyzer. The VNA was initially calibrated using a Keysight 85221A mechanical calibration kit with open, short, through, and load testing capabilities. After calibration, the VNA was set to 0 dBm power and 300 Hz bandwidth to measure transmission gain (S_21_) over 4001 equally spaced data points between 1 and 5 GHz.

## Results and discussion

**Figure 3:**
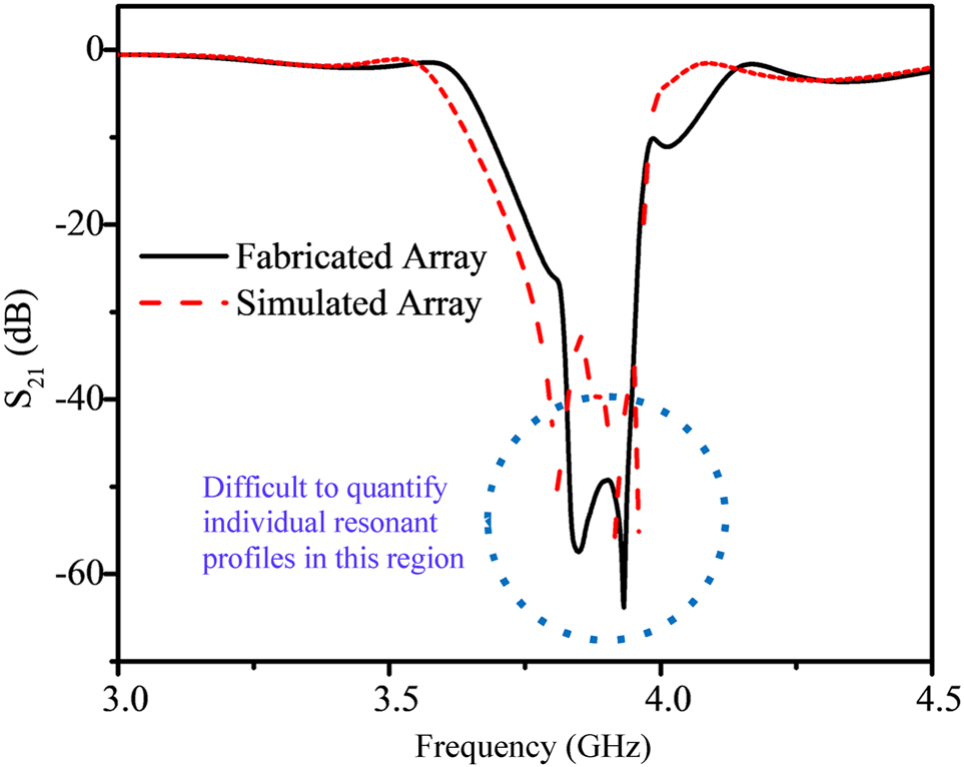
Simulated and fabricated S_21_ response of the resonator array highlighted the difficulty in isolating and analyzing individual resonant profiles because of multiple resonant responses overlapping at roughly the same frequency. Simulated results were generated from design using *HFSS* 19.0 (https://www.ansys.com/).

To demonstrate the effectiveness of the sensor array, its tunability was first investigated as a method of selecting individual resonators using liquid metal. The S_21_ profiles of the simulated and fabricated resonator recorded under initial operating conditions: with the microfluidic channel but without liquid metal tuning, are shown in Fig. 3. The S_21_ response consisted of six overlapping resonant profiles, one from each of the resonators in the array. The resonant frequency was measured to be between 3.8 and 4.0 GHz, the 200 MHz difference was mostly attributed to the bandwidth of the individual resonant response (~ 60 MHz) as well as minor differences in resonator position and resonator-to-resonator coupling.

Based on the results of Fig. 3, the response of the fabricated resonator closely matched the simulated resonator response although it was apparent from their similar resonant frequencies that analysis of any single resonator’s response would be difficult. The sensor in this form could not adequately characterize changes in the resonant amplitude and frequency without using peak modelling or fitting algorithms. Because microwave resonator sensing is based on quantifying these changes, it was necessary to isolate the response of an individual resonator for analysis and so liquid metal was introduced.

**Figure 4:**
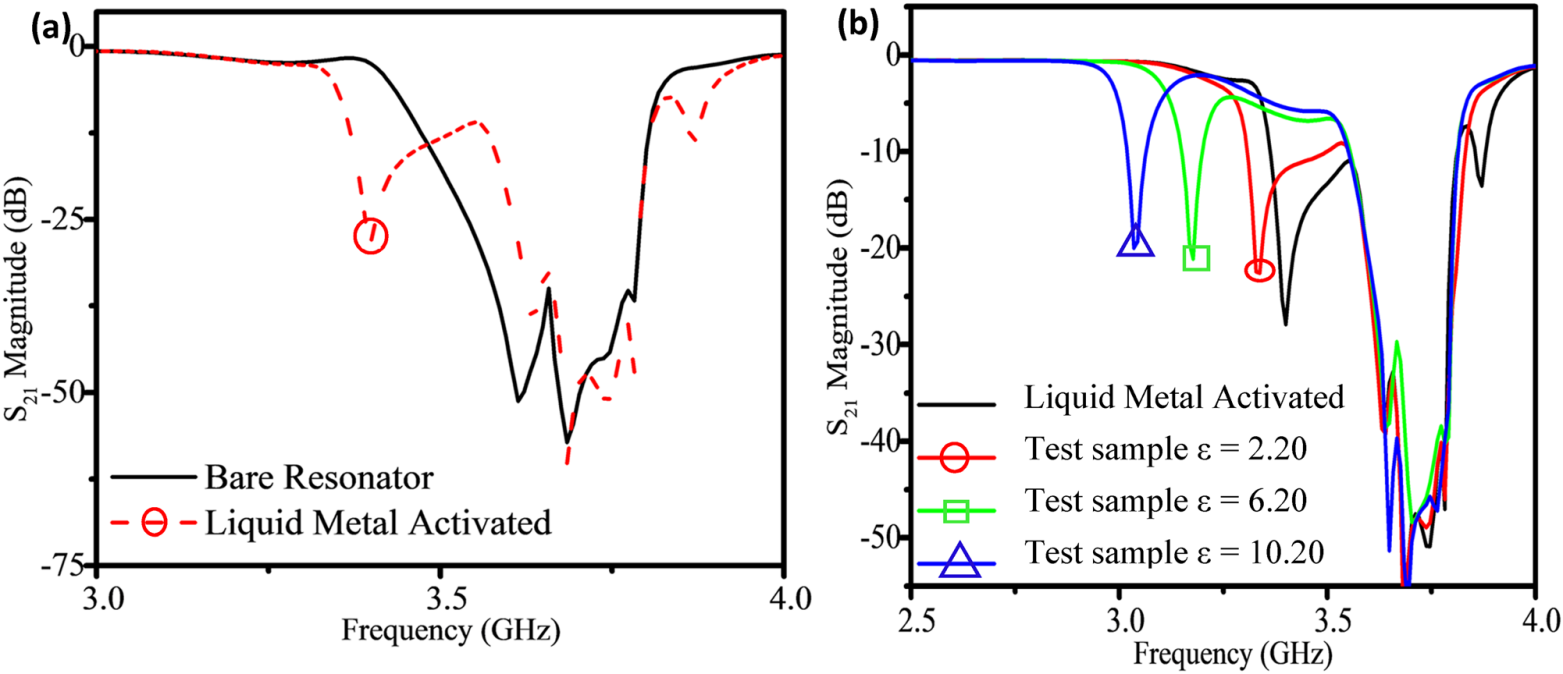
(**a**) Simulation results illustrated that liquid metal reduced the resonant frequency of the selected resonator (red, circled), isolating its response from the other resonators for easier analysis and (**b**) sensing performance was measured with the isolated response for test samples with a dielectric permittivity of 2.2, 6.2, and 10.2.

To demonstrate the ability of liquid metal to tune the simulated array sensor and isolate specific resonator responses for analysis, test materials with a relative permittivity of 2.20 were placed over the sensing gap of every resonator and the results are shown in Fig. 4a. The *S*_21_ response shifted identically irrespective of the resonator tested, demonstrating consistent sensitivity of each resonator in the array structure due to their identical dimensions. However, since this similarity in response made analysis more difficult, 2 cm of liquid metal (simulated as a perfect electrical conductor) was positioned above the selection gap of resonator *A*. *HFSS* simulation also introduced test materials with different permittivity (2.2, 6.2, 10.2) over the sensing gap of the selected resonator, as shown in Fig. 4b, which confirmed the sensitivity of the selected resonator to test materials while the response of the other non-selected resonators was unchanged.

The simulated array with no liquid metal demonstrated a resonant frequency and amplitude of 3.75 GHz and − 67 dB, respectively, while introducing the liquid metal over a single resonator tuned its frequency to 3.3 GHz with an amplitude of − 27 dB. The simulated frequency tuning range of almost 600 MHz was more than sufficient to separate the selected resonator response from the other responses. As dielectric materials were placed above the sensing gap, *G*_2_, the impact of the materials was observed at the isolated notch response with a sensitivity of 30 MHz per unit permittivity. The isolated *S*_21_ response could be associated to the array position of the liquid metal due to the resonant frequency of resonator *A* being tuned, effectively enabling the fluidic channel to act as a multiplexer. In cases where different measurements were performed at each of the resonator locations, liquid metal could be used to probe each response without requiring changes to the mechanics or electronics of the system.

**Figure 5:**
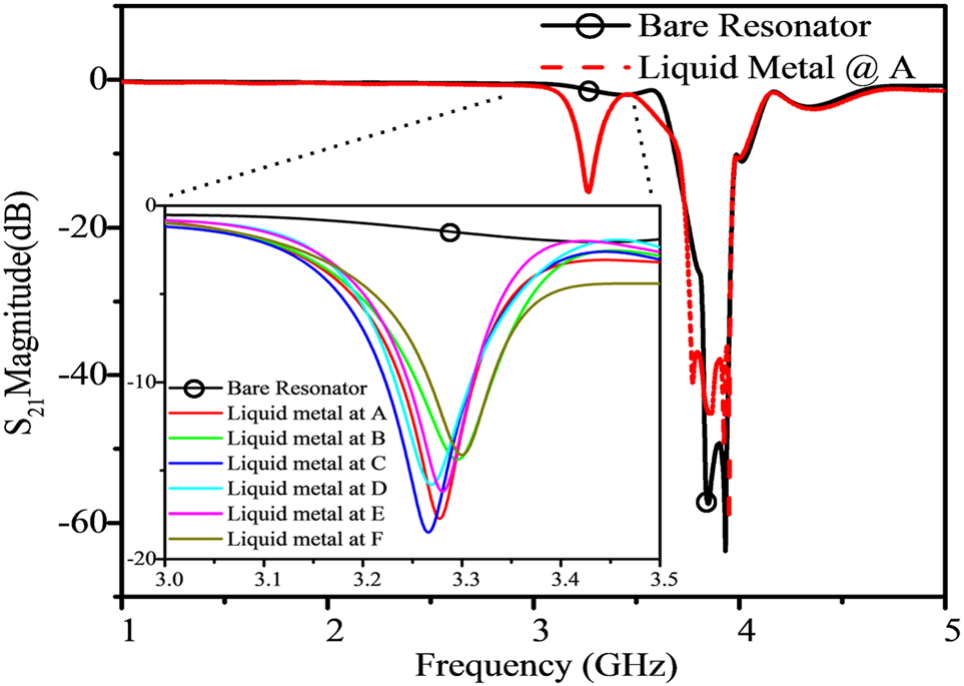
The fabricated resonator with and without liquid metal loading at resonator A, inset shows each tuned resonator had a resonant frequency and amplitude response between 3.26 and 3.29 GHz and − 18 to − 15 dB, consistent among all 6 resonators.

The fabricated sensor was tested with an experimental procedure which matched the simulation settings; the tuning performance of the liquid metal was evaluated by filling the channel with a 2 cm length of liquid metal and positioning it above the selection gap, G_1_, of the targeted resonator. Figure 5 compares the *S*_21_ response of the fabricated sensor with no liquid metal with the *S*_21_ response when liquid metal is placed separately on resonators *A* through *F*.

Based on the results presented in Fig. 5, liquid–metal loading of the fabricated resonators showed consistent tunability to create a 3.28 GHz notch with ~ 600 MHz tuning range from the other resonators’ responses, which was sufficient to be distinguished from the main frequency of 3.9 GHz. This tuning effect occurred almost identically for every resonator selected within the array, with the resulting resonant frequency of each selected resonator measuring within 3.26 and 3.29 GHz (a tuning variation of 30 MHz) and the resulting resonant amplitude between − 15 to − 18 dB. Thus, the tuning process including the Galinstan and hydrocal oil allowed for large capacitive loading to be transferred along the circuit via microfluidic action, offering controlled isolation of selected resonator profiles, in a repeatable and reversible process. Any variation in the isolated response was most likely due to slight differences in liquid metal alignment and multi-resonator coupling, but did not affect the sensitivity measured when test materials were introduced.

**Figure 6:**
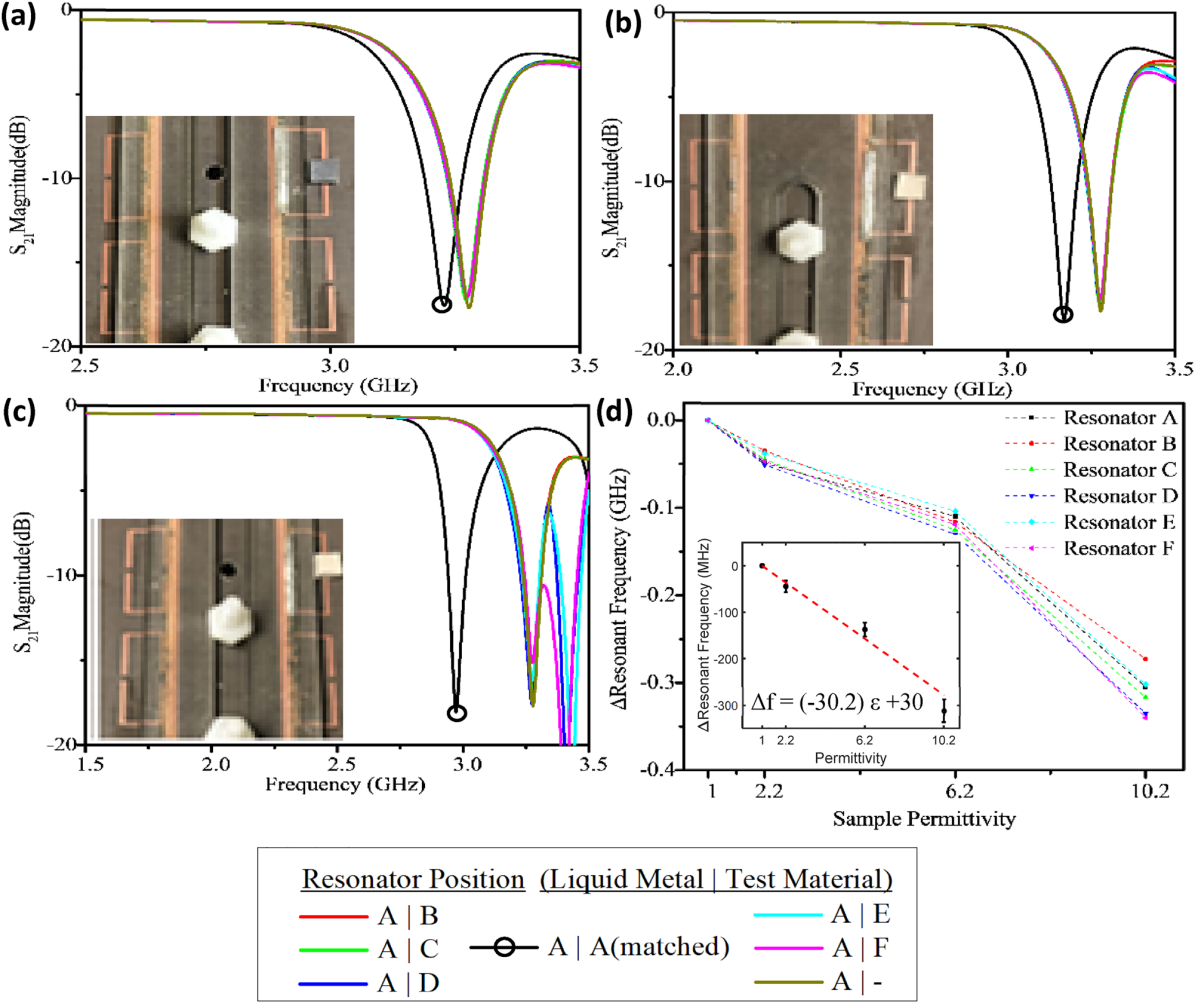
The fabricated sensor’s frequency response when resonator *A* is selected by the liquid metal (inset photos). Test materials with permittivity of 2.2 (**a**), 6.2 (**b**), and 10.2 (**c**) induced a measured frequency shift when placed on the selected resonator (circled black). The recorded sensitivity curves are shown for each individual resonator (**d**) and error bars of the inset generated by performing each test three times.

After successfully using liquid metal to tune the resonator, the sensing capabilities of the array were investigated. The second ring gap which was not covered by the microfluidic channel was used as a sensing region that could now have its response isolated by manipulating the liquid metal position, enabling simplified analysis. Sensing experiments were performed by positioning the liquid metal above resonator *A* to create the distinguishable resonant notch (as shown in Fig. 6). Then, test materials from Rogers™ with relative permittivity of 2.20, 6.20, and 10.20 and dimensions 5 × 5 × 0.79 mm^3^ were placed one at a time on the sensing gap of each resonator, *A* through *F*, and the *S*_21_ response of the array was recorded in Fig. 6a–c. All experiments were performed in ambient lab conditions: 23 °C and 35% relative humidity to ensure environmental effects on the covered resonator were mitigated. Sensitivity experiments were performed in triplicate to generate an accurate sensitivity curve for the sensor array. To determine the long-term stability of the sensor, particularly the hydrocal oil and liquid metal within the channel, tests were performed 2 months after the original experiments and no visible signs of reaction or decomposition were observed, and the tuning range or tuning variation were not beyond the bounds of the original results.

Importantly, both the test material and liquid metal needed to be on the same resonator for sensing to occur without signal overlap which was only possible due to the strategic tuning of individual resonators with capacitive loading. These resonators acted independently, meaning the isolated resonant response did not change when test materials were introduced on any of the unselected resonators—such as loading resonator *A* with liquid metal but adding the test material at resonator *B* through *F*. These sensing experiments were also performed in turn by selecting the other resonators (*B* through *F*) with liquid metal while the change in resonant profile of each activated resonator was measured with all three sample materials, their sensitivity was found to be very consistent (Fig. 6d). The resonant frequency sensitivity of all the resonators was measured to be between 29 and 33 MHz per unit *ε* when selected by liquid metal, roughly in line with other current dielectric resonator sensors^31,32^.

The resonator sensors performed with comparable sensitivity to other state-of-the-art resonator sensors, as shown in Table 1. The table compares other works sensitivity to different dielectric materials as an absolute shift (MHz) as well as a relative (%) change in resonant frequency. Resonator size and tunability were also compared as they are important parameters of an effective and functional sensor.

**Table 1:** Sensitivity comparison of state-of-the-art resonator sensors.

Design	Sensitivity @ resonant frequency	Relative sensitivity (%)	Resonator dimensions	Tunable	References
Microstrip sensor for low ε materials	600 MHz/ε @ 15.2 GHz	3.9	1 cm × 1 cm	No	^33^
Extended gap SRR	7 MHz/ε @ 1.95 GHz	0.4	3 cm × 3 cm	No	^34^
Modified SRR (passive)	50 MHz/ε @ 2.1 GHz	2.4	2 cm × 4 cm	No	^35^
Tri-band resonator	80 MHz/ε @ 2.5 GHz	3.2	4 cm × 8 cm	No, multiple resonator	^15^
MEMS-based tunable resonator	–	–	4.5 cm × 1.5 cm	Yes, 1400 MHz	^23^
Multi-band magnet selected resonator	300 MHz shift in response to magnetic field	–	1.5 cm × 4 cm	Yes, with magnetic switching	^36^
Liquid metal resonator array	30 MHz/ε @ 3.3 GHz	0.9	1 cm × 2 cm	Yes, 600 MHz	This work

Considering their unique design with liquid metal and the inclusion of a microchannel for resonant frequency tuning it is intriguing that sensitivity is still promising and, as the results show, still capable of distinguishing many different materials using this method. The resonator size was not altered to accommodate the channel and due to the array structure the overall resonator size per tested sample is much smaller because only one transmission line was required.

Due to the identical dimensions of each resonator, sensing of all six resonators in the array could be performed over a narrow band frequency range of 3.30–2.80 GHz because each resonator did not require a unique frequency bandwidth once selected. Instead, sensing of numerous materials or inputs can occur over the same frequency range simply by selecting different resonators with Galinstan as required. This can enable sensing multiple components within complex systems using a compact design and a narrow frequency range.

## Conclusion

A modified split ring resonator array with a liquid metal tuning method was presented to provide individual resonator selection within an array, leading to simplified signal analysis. The developed sensor enabled measuring the resonant frequency and amplitude of multiple resonators over the same frequency span, reducing the frequency spacing and bandwidth required for sensing applications. By testing the resonator under different configurations, it was found that the test material could only be sensed when placed on a selected resonator and that each resonator had a sensitivity of 30 MHz per unit ε, with less than 3 dB variation in the resonant amplitude. It was also shown that sensing was unaffected by materials on unselected resonators, exhibiting the independent operation of each resonator within the array and the utility of position-sensitive liquid metal selection. The application of the proposed structure could be fully realized with integration of chemically sensitive smart materials, as each resonator could respond to a different environmental variable: temperature, humidity, pH, or pressure to name a few. With that in mind, the improved control, reduced bandwidth requirements, and individual resonator selection offered by liquid metal shows potential to improve the efficiency and effectiveness of modern wireless sensors.
